# MFMINet: Multimodal fusion and cross-layer interaction network for semantic segmentation of high-resolution remote sensing images

**DOI:** 10.1016/j.isci.2026.114705

**Published:** 2026-01-16

**Authors:** Junjie Chen, Feiting Wang, Jiangtao Fang, Yu Zhu, Yu-an Zhang, Qiongqiong Hu

**Affiliations:** 1 School of Computer Technology and Application, Qinghai University, 251, Ningda Road, Xining, Qinghai 810016, China; 2 Qinghai Provincial Laboratory for Intelligent Computing and Application, Qinghai University, 251, Ningda Road, Xining, Qinghai 810016, China; 3 School of Computer Science, Northwestern Polytechnical University, No. 1, Dongxiang Road, Xi’an, Shaanxi 710129, China

**Keywords:** earth sciences, remote sensing, machine learning

## Abstract

In recent years, semantic segmentation of remote sensing images using deep convolutional neural networks (CNNs) has seen rapid development in fields like urban planning and land cover analysis. However, reliance on a single imaging modality is often hampered by spectral ambiguity, the absence of elevation cues, and geometric confusion, limiting the discrimination of spectrally similar yet distinct categories like roads versus roofs. While multisource data fusion has emerged as a promising solution, effectively leveraging complementary information from multimodal features remains challenging. To address these challenges, we propose a multimodal fusion and multilayer interaction network (MFMINet), a two-way encoder-decoder network. Our model employs a multimodal cross-layer fusion module (MCFM) to integrate high-level semantic information with low-level spatial details, exploring the complementarities between different information modalities. Additionally, we introduce a self-attention module (SAM) to capture long-range spatial dependencies and refine fused features. Additionally, we develop a feature enhancement module (FEM) that intelligently selects between Transformer blocks for narrow channels and CNN blocks for wide channels, followed by point-wise convolution for optimal feature integration. Furthermore, we propose a dual spatial awareness module (DSAM) to mitigate downsampling effects and process global multiscale contextual information. Extensive experiments on ISPRS Vaihingen and Potsdam datasets demonstrate superior performance, with mIoU reaching 89.96% and 88.24%, respectively, validating the effectiveness of our method.

## Highlights

• MFMINet enables effective fusion of IRRG and nDSM, boosting segmentation accuracy
• MCFM module strengthens fusion of IRRG and nDSM information
• SAM and DSAM significantly improve global and multiscale context modeling
• FEM module intelligently selects transformer or CNN for efficient feature aggregation

## Graphical abstract

**Figure:**
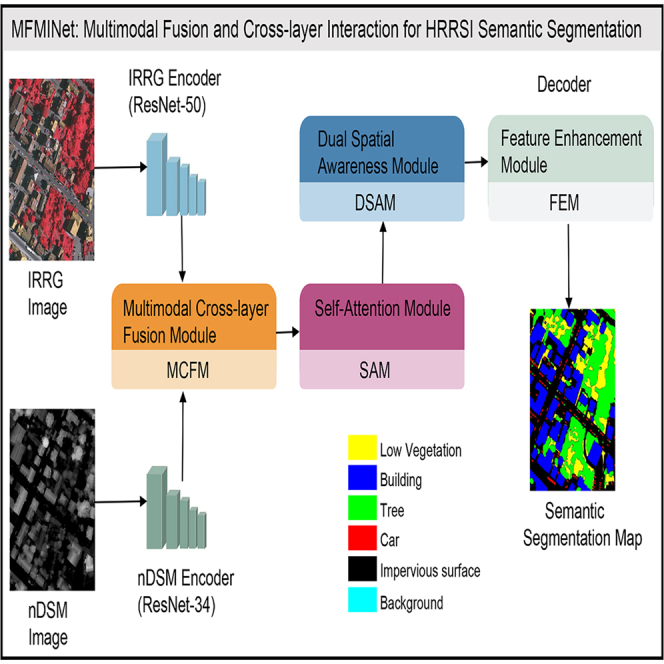

## Introduction

In the field of computer vision (CV), semantic segmentation is one of the fundamental tasks, which has been widely studied in the field of remote sensing.^1^ Semantic segmentation of high resolution remote sensing images aims to assign a label to each image pixel. With the application of deep convolutional neural networks (DCNNs) in the field of CV, semantic segmentation for HRRSI (high resolution remote sensing image) has attracted a lot of attention and can be used in a variety of fields, such as road extraction,^2^ point cloud segmentation,^3^ building segmentation,^4^ map services,^5^^,^^6^ and urban planning.^7^

Currently, there are still many challenges for the task of segmenting ultra-high resolution remote sensing images of urban scenes. In urban remote sensing images, various issues such as light transformations, mutual occlusion of objects, and shadows can adversely affect the results of semantic segmentation. In addition, there are similarities between different classes of objects (e.g., low vegetation and trees), which makes the model prone to confuse these objects. In the past, DCNN was usually applied to traditional RGB (red-green-blue) high resolution remote sensing image segmentation tasks, such as MAResUnet.^8^ Later, researchers tried to apply transformer to HRRSI semantic segmentation, taking advantage of transformer’s self-attention machine and the advantages in making and capturing long-range dependencies.^9^ The advantages of transformer have achieved good results. However, the HRRSI semantic segmentation task requires labeling each pixel and assigning each pixel to a class, which may lead to the problem of intraclass and interclass inconsistencies due to the fact that each pixel has rich detail information including color, texture, and so forth.^10^ In the semantic segmentation driven by the HRRSI deep neural network, each pixel needs to be accurately labeled, and this step tends to class consistency and disparity problems. These problems are usually due to the limited receptive field of the convolutional kernel, which fails to adequately capture the overall semantic context. To address this problem, downsampling strategies, such as pooling, are often adopted to expand the receptive field. Although this helps to extract semantic information, it is also accompanied by the loss of spatial details in the image. Therefore, how to balance the fusion of high-level semantic information and low-level spatial information in the semantic segmentation process and reduce the spatial resolution blurring caused by downsampling is a technical challenge currently faced.

It is worth mentioning that the field of CV has faced a similar challenge as that of remote sensing. In order to solve this challenge, some scholars in the field of CV have proposed the visual transformer (Vit),^11^ which exploits the self-attention mechanism in the transformer,^12^ which enables the model to simultaneously consider the relationships between all pixels in the feature map, thus enhancing the model’s modeling of global contextual information. In addition, unimodal semantic segmentation has achieved excellent performance with the support of some powerful backbone networks. However, since HRRSI is usually characterized by complex scenes, objects with multivariate features and high intraclass variance and interclass similarity. This makes it more difficult to distinguish regions with similar spectral features using only unimodal data. Recent studies have shown that multimodal data can show the feature information of the target from different angles,^13^ so the complementary nature of multimodal data can be used to better distinguish regions with similar spectral features.

The use of multimodal data fusion in RGB-D (RGB and depth) images for semantic segmentation is a more general approach,^14^ which enhances spatial details in RGB image features by introducing a depth map. By combining the feature information of visual images with the spatial information in the depth map, RGB-D semantic segmentation thus achieves high performance that cannot be easily achieved by methods that perform semantic segmentation using only visual information. However, since HRRSI contains unique spectral combinations, usually contains small objects and complex scenes, and the elevation information contained in the normalized Digital Surface Model (nDSM) is complex and noisy, in addition, most existing semantic segmentation models only use element-addition or channel-by-channel concatenation, which fails to efficiently utilize the complementary feature information in multimodal data to enhance model performance. Therefore, RGB-D semantic segmentation is not directly applicable to HRRSI semantic segmentation when infrared-red-green (IRRG) images are used in combination with nDSM images in HRRSI semantic segmentation.

**Figure 1:**
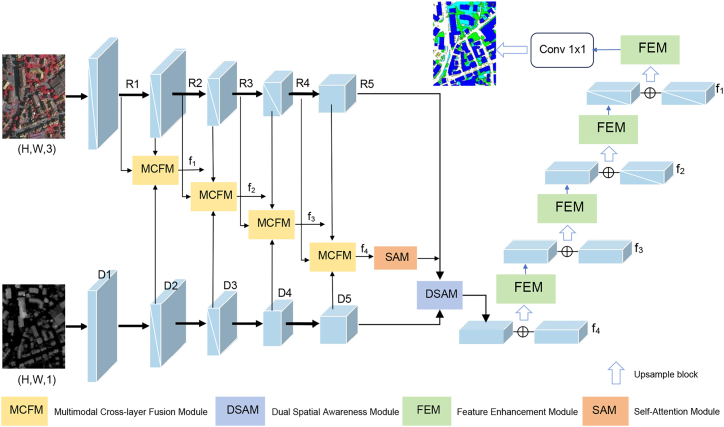
The overall structure of MFMINet The schematic illustrates the two-branch encoder-decoder network designed for multimodal remote sensing image segmentation. It processes an IRRG image and an nDSM through separate backbones, fuses features via the MCFM modules, refines context with the SAM and DSAM modules, and reconstructs the prediction using the adaptive FEM module in the decoder.

**Figure 2:**
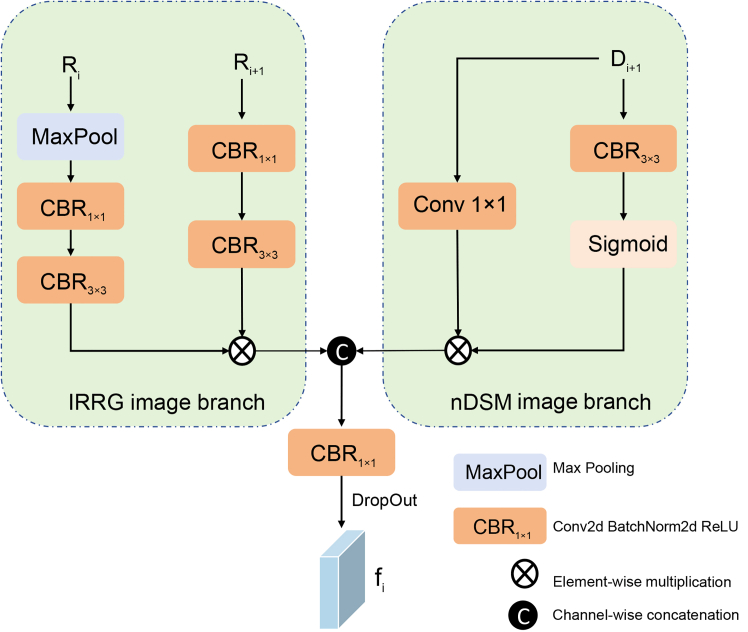
Multimodal cross-layer fusion module The diagram details the MCFM module, which integrates features from different modalities (IRRG and nDSM) and network layers. It shows the parallel processing paths for each modality, the element-wise multiplication for focused fusion, and the final concatenation and regularization step.

**Figure 3:**
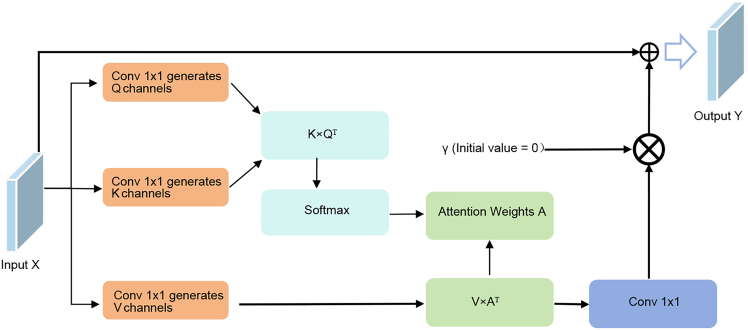
Self-attention module The diagram depicts the SAM module, which captures long-range dependencies. It visualizes the transformation of input features into Query (Q), Key (K), and Value (V) vectors, the computation of the spatial attention matrix, and the generation of the attended output feature map.

**Figure 4:**
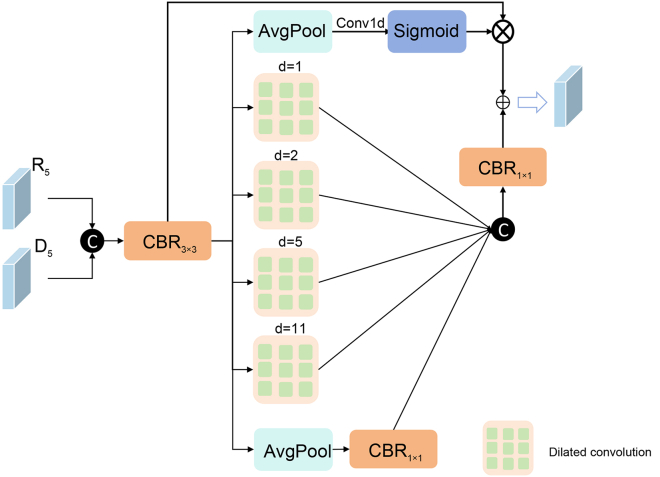
Dual-way spatial awareness module The diagram shows the DSAM module, which extracts multiscale contextual information. It presents the six parallel processing branches, including a channel attention branch, four dilated convolutional branches with different rates, and a fusion branch that aggregates the multiscale features.

**Figure 5:**
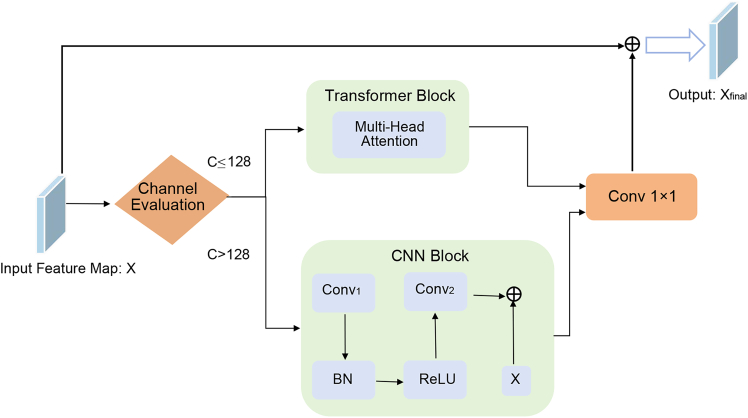
Feature enhancement module The flowchart illustrates the FEM’s adaptive mechanism. It demonstrates the decision process based on channel width: using a transformer block for narrow-channel features or a CNN block for wide-channel features, followed by point-wise convolution for integration.

In order to be able to effectively utilize feature information from nDSM data for accurate semantic segmentation of HRRSI, we propose a two-way encoder-decoder network called multimodal fusion and multilayer interaction network (MFMINet), as shown in Figure 1. Specifically, in this paper, we develop a two-branch model that takes into account the differences between different layer features and different modality features, and our proposed model passes through a multimodal cross-layer fusion module (MCFM) (Figure 2) in order to fuse high-level semantic information with low-level spatial detail information. In addition, complementary cross-modal features are extracted for fusion. To enhance global context modeling capabilities, we introduce a self-attention module (SAM) (Figure 3) that captures long-range spatial dependencies and refines the fused multimodal features before multiscale processing. Next, we pass the proposed dual spatial awareness module (DSAM) (Figure 4) in order to process the global multiscale contextual information and provide guidance for subsequent decoding. Finally, the shallow and deep features obtained in the encoder are fed into a decoder where we propose a feature enhancement module (FEM) (Figure 5) in order to fuse the features at all levels and filter out redundant information. The FEM intelligently selects between transformer blocks for narrow channels and CNN blocks for wide channels, followed by point-wise convolution for optimal feature integration, while further refining the features to enhance semantic segmentation. The contributions of this work are 3-fold and are summarized as follows.

1. A network model MFMINet for cross-modal semantic segmentation of IRRG images using nDSM is proposed, which retains the advantages of IRRG images and effectively fuses complementary information to facilitate the model’s learning of semantic information.
2. We propose an MCFM module to fully integrate nDSM and IRRG multimodal information. Additionally, we introduce SAM module to capture long-range spatial dependencies and enhance global context modeling of the fused features. Furthermore, we use DSAM module to extract multiscale contextual features by dual-path interaction and incorporating a channel attention mechanism, which establishes relationships between features at different scales.
3. We propose a FEM module for fusing the features extracted by the encoder and decoder for efficient feature aggregation. The FEM intelligently selects between transformer blocks for narrow channels (≤128) and convolutional neural network (CNN) blocks for wide channels (>128), followed by point-wise convolution for optimal channel information integration.

## Results and discussion

### Related work

#### Single-modal semantic segmentation

In recent research, with the advancement of big data technology and its profound impact on the field of CV, remote sensing data have become easily and publicly available for various applications, such as the ISPRS Vaihingen and Potsdam datasets, the ESA Sentinel 1/2 data, and the UAVid data. Fully Convolutional Networks (FCNs),^15^ as a representative encoder-decoder network that replaces the fully connected layer in traditional CNNs, enabling the network to process inputs of arbitrary size and output results of corresponding size. By jumping connections, FCN combines the feature maps of different layers and retains more spatial detail information. On this basis, U-Net^16^ proposes a more efficient skip connection structure, which achieves the precise fusion of different feature maps and improves the segmentation accuracy. SegNet^17^ makes the decoding process more standardized by recording the pooling indexes during the encoding process and using them for the supervision of the decoding process. Dilated convolution is commonly used to alleviate the contradiction between the feature map and the size of the receptive field.^18^ It uses a zero convolution kernel to convolve the input image, which enables to obtain feature maps of different scales while ensuring that the receptive field is not reduced, thus obtaining more contextual information. Representatives of image segmentation developed using this theory are DeepLabv3+,^19^ which in its turn uses an optimized hybrid strategy and combines an encoder-decoder architecture with spatial pyramid pooling (ASPP) techniques. By adjusting the dilated convolution rate, the method strikes a good balance between the resolution of the encoder feature map, segmentation accuracy, and computational efficiency. However, scale transformations and ground objects with high similarity features^20^ pose new challenges for semantic segmentation of remote sensing images. Recognition of these complex targets requires finer spatial features and global contextual information. To address the problem, existing methods apply multiscale and attention mechanisms to model long-range dependencies and feature refinement. A2-FPN^21^ learns multiscale features through a feature pyramid network and enhances multiscale feature learning with an attention aggregation module (AAM). Li et al.^22^ designed a multistage attention reconstruction network by adding a linear attention mechanism to the skip connections of the original UNet to send where long-range dependencies. Zhao et al.^23^ proposed a semantic segmentation network with an attention mechanism that uses a pyramid attention pooling module for adaptive feature refinement. In addition, to reduce the workload of image annotation, Hua et al.^24^ proposed semantic segmentation based on incomplete annotation and regularization of feature-space relations to complement the supervision. Wang et al.^25^ proposed a two-path sparse hierarchical network that involves rich cross-scale feature interactions and aggregation of multilevel features to reduce semantic and spatial gaps between features.

#### Multimodal fusion for semantic segmentation

The integration of complementary information from multiple data sources has become a pivotal strategy to overcome the limitations of single-modality analysis, such as spectral ambiguity or the lack of geometric and thermal context. This section reviews fusion methodologies across different modalities, with a focus on RGB-Depth (RGB-D) and RGB-Thermal (RGB-T) segmentation, categorizing them by their fusion strategy to provide a coherent technical landscape.

Early and Feature-Level Fusion: A foundational approach is to fuse information at the input or encoder level. Pioneering work by Noh et al.^26^ introduced FuseNet, which processes RGB images and depth maps through twin encoders and fuses their intermediate features via element-wise addition. Extending this concept beyond depth, Ha et al.^27^ proposed MFNet, a real-time, two-branch network for RGB-T segmentation in urban scenes. Similarly, Liu et al.^28^ improved HHA encoding and performed weighted addition on features from RGB-D branches, while Shreyas et al.^29^ developed a two-branch CNN that processes RGB and thermal features independently before combination.

Decoder-Focused and Hierarchical Fusion: To better recover spatial details, several methods emphasize fusion during decoding or across multiple network levels. Wang et al.^30^ designed a deconvolutional network where RGB and depth data are processed in the encoding and decoding phases, respectively. Sun et al.^31^ proposed the FuseSeg network, where features initially fused by element-wise addition are subsequently fused in the decoder through tensor concatenation to mitigate information loss. Dutta et al.^32^ implemented explicit hierarchical fusion in a multilinear network, merging independently encoded RGB and thermal features at multiple decoding levels.

Attention and Advanced Interaction Mechanisms: Recent trends leverage attention and specialized modules for adaptive fusion. Zhou et al.^33^ proposed a three-stream SAM with asymmetric input and cross-modality filtering streams for sophisticated feature fusion. In the RGB-T domain, Zhou et al.^34^ generated prior edge maps and embedded this structural information into the feature maps. Other works have integrated multiscale context modules, such as the spatial pyramid pooling in Dai et al.’s ResFusion network,^35^ or efficient encoder-decoder architectures like the ESANet by Seichter et al.^36^ and RTFNet by Sun et al.^37^

Despite these advancements, a common limitation among many existing methods is the reliance on a single or simplistic fusion strategy, which can lead to the under-utilization of distinctive features across different levels of abstraction. Our proposed method is designed to address this gap by introducing a more flexible and comprehensive fusion framework.

#### Multimodal HRRSI semantic segmentation

In the research in the field of remote sensing images, pixel-level multisource remote sensing data fusion is mainly based on optical remote sensing images, nDSM, Light Detection and Ranging (LiDAR), and Synthetic Aperture Radar (SAR). Semantic segmentation of multimodal remote sensing images can integrate different types of the same feature to obtain high-quality information for tasks such as change detection. Zhao et al.^38^ proposed the MS2-NET network, which is designed with a multistage fusion module to calibrate the bias information by filtering the noise from the multimodal data. In addition, similar feature points are aggregated by the proposed multisource attention thereby enhancing the distinguishability of features with different modalities. Sun et al.^39^ proposed a multimodal fusion mechanism as well as multistage feature fusion modules through which adaptive fusion of multimodal features can be achieved to enhance global to local contextual fusion. Xiong et al.^40^ proposed a transformer-based intermediate multimodal fusion module, which uses intermediate learnable tokens to fuse RGB and high-level modality features through a self-attention mechanism.

Further advancing this field, several state-of-the-art (SOTA) multimodal fusion methods have been proposed, which are also used as baselines in our comparative experiments. CMFNet^41^ fuses multiscale convolutional feature maps of optical remote sensing imagery and digital surface model data through a cross-modal multiscale transformer and a multiscale context augmentation transformer to effectively extract multiscale contextual information and long-range dependencies. CMGFNet^42^ uses a residual-like depth-separable convolution (R-DSC) to improve the performance of the upsampling process and to reduce the parameter and time complexity of the decoder part. MGCNet^43^ employs a gating unit for each layer of the fused information to reduce inter-layer differences while enhancing layer-specific uniqueness. SFANet^44^ introduces an adaptive feature aggregation module that can self-adaptively adjust the weights of feature fusion according to the importance of different feature layers. Finally, FTransUNet^45^ combines the advantages of the transformer architecture with the encoder-decoder structure of U-Net, leveraging transformer’s powerful feature extraction and long-range dependency modeling capabilities within an efficient upsampling framework.

### Experimental settings

#### Dataset

We conducted experiments on two public HRRSI benchmark datasets, Vaihingen and Potsdam, which are provided by the International Society for Photogrammetry and Remote Sensing (ISPRS) and are more commonly used in the remote sensing field. Both Vaihingen and Potsdam datasets have six categories, namely impervious surface, building, low vegetation, tree, car, and background.

1. The Vaihingen dataset is a small village in Germany with a large number of individual and small buildings. It contains 33 images with a spatial resolution of 9 cm, and we choose images from the IRRG spectrum. The image size of this dataset is variable, with an average size of 2494 × 2064 pixels.
2. The Potsdam dataset is a typical historical city with a continuous and large architectural complex. This dataset contains 38 images with a spatial resolution of 5 cm, and we still choose images from the IRRG spectrum. The image size of this dataset is 6000 × 6000 pixels.

**Figure 6:**
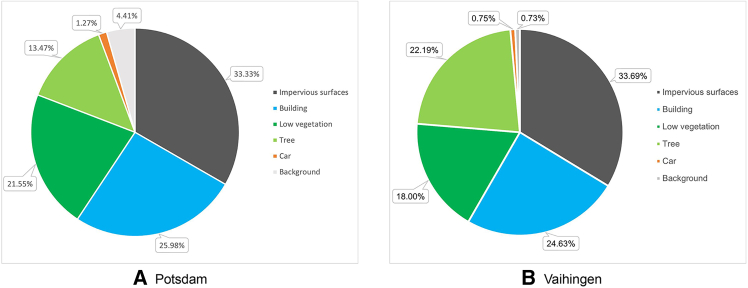
Proportion of the number of pixels in each category in Potsdam and Vaihingen remote sensing datasets Bar charts display the pixel proportion for each semantic class in the (A) Potsdam and (B) Vaihingen datasets. The graphs highlight the significant class imbalance present in both benchmarks, such as the low proportion of “Car” and “Background” pixels.

Since the datasets used in this paper contain images of larger size, we cropped the images of both datasets to 256 × 256 pixels using a non-overlapping sliding window. This process resulted in 10,113 training and 2398 validation images for the Vaihingen dataset, and 40,627 training and 10,157 validation images for the Potsdam dataset. In addition, horizontal flipping, random rotation, and addition of noise were applied to the training images to enhance the data. The proportion of pixels in each category to the total number of pixels in the two datasets is shown in Figure 6, and it can be seen that the two datasets are characterized by an imbalance of categories. For example, in the Potsdam dataset, pixels belong to the background and car categories account for only 4.41% and 1.27% of the total number of pixels, respectively, which is significantly lower than the other categories.

#### Implementation details

ResNet-50 is used as the backbone network for IRRG image processing in MFMINet. Considering that the feature information to be extracted from nDSM images mainly includes elevation information, we use ResNet-34 as the backbone network for processing nDSM images. In this paper, the experimental equipment is Ubuntu 20.04, the GPU is NVIDIA GTX1080Ti GPU, and the RAM is 12 GB. The model running environment is PyTorch 2.0.1 and Python 3.10.11. The Adam optimizer is used, with the weight decay set to 0.0001 and the initial learning rate set to 5e-4. During the training period, the maximum number of iterations is set to 100. In each iteration, a batch of data with a size of 16 is taken for training until all training images in the dataset have been processed, which completes one round of iteration.

#### Evaluation metrics

We use common evaluation metrics in semantic segmentation, including precision, recall, F1 score, and mean intersection over union (mIoU), to evaluate the performance of our proposed method. Among them, we decided to use the mIoU as the main evaluation criterion. The mIoU is the intersection of the true and predicted values of a pixel divided by the concatenation of the true and predicted values of the pixel, which gives a good indication of how well a category is extracted. These evaluation metrics are computed as follows.

(Equation 1) $$recall=\frac{TP}{TP+FN}$$

(Equation 2) $$precision=\frac{TP}{TP+FP}$$

(Equation 3) $$F1=\frac{2\times precision\times recall}{precision+recall}$$

(Equation 4) $$mIoU=\frac{1}{n}\sum_{i=1}^{n}\frac{TP}{\sum_{j=1}^{n}\left(FN+FP\right)-TP}$$

where TP (True Positive) indicates that the actual pixel is positive, and the prediction is also positive, that is, the prediction is correct. TN (True Negative) means that the actual is negative, and the prediction is also negative. FN (False Negative) means that the prediction was false, but it was actually true. FP (False Positive) means that the prediction was positive, but it was actually negative.

### Comparative experiments and discussion

Our proposed model MFMINet is compared with some classical and SOTA semantic segmentation methods, including PSPNet,^46^ DeepLabV3+,^19^ SegNet,^17^ FCN-8s,^15^ CMTFNet,^47^ CMFNet,^41^ CMGFNet,^42^ MGCNet,^43^ SFANet,^44^ and FTransUNet.^45^ Among them, PSPNet, DeepLabV3+, SegNet, FCN-8s, and CMTFNet are unimodal semantic segmentation methods, while the rest of the methods use multimodal semantic segmentation methods. FCN is one of the basic frameworks for semantic segmentation, and it is the first full convolutional network that provides end-to-end prediction of an image on the pixel level. SegNet, which is a semantic segmentation network based on FCN, makes use of the indexing information in the pooling process to perform accurate upsampling and thus improve the segmentation accuracy. The proposed Pyramid Scene Parsing Network aggregates contextual information based on different regions to mine global contextual information. DeepLabv3+ is a deep learning model used in the field of semantic segmentation of natural images. The model is designed based on an encoder-decoder architecture, which effectively captures multiscale contextual information through spatial pyramid pooling technique (ASPP) and performs fine-grained segmentation processing at the object edges. CMTFNet is a deep-learning model for semantic segmentation of remote-sensing images. Its core idea is to fuse CNNs with a multiscale transformer architecture, simultaneously capturing local spatial details and global contextual information. The multimodal methods employed for comparison have been detailed in the multimodal HRRSI semantic segmentation section. They represent a range of advanced fusion strategies, including cross-modal transformers (CMFNet and FTransUNet), efficient decoding designs (CMGFNet), and adaptive or gated fusion mechanisms (MGCNet and SFANet). We directly employ these published models to ensure a fair and reproducible comparison.

#### Experiments on Vaihingen dataset

We assessed the semantic segmentation capabilities of various cutting-edge techniques on the ISPRS Vaihingen dataset. The findings presented in Tables 1 and 2 reveal the performance of these methods across different land cover classes. Our proposed method, MFMINet, achieved an mIoU score of 89.96%, surpassing the second-best method, SFANet, by 1.20%. As depicted in Table 2, MFMINet excels in all categories, especially in buildings, trees, and low vegetation, with mIoU scores of 96.04%, 90.57%, and 88.73%, respectively. This underscores the effectiveness of MFMINet in capturing intricate details and object boundaries, which is vital for remote sensing applications.

**Table 1:** Results evaluated by recall (%), precision (%), F1 (%), and mIoU(%) on the Vaihingen dataset

Method	Modality	Backbone	Recall	Precision	F1	mIoU
DeepLabV3+	Uni	ResNet	93.42	92.38	92.90	86.95
PSPNet	Uni	ResNet	92.56	91.20	91.87	85.34
SegNet	Uni	VGG	91.73	91.30	91.51	84.70
FCN-8s	Uni	VGG	91.90	91.28	91.59	84.76
CMTFNet	Uni	ResNet	93.78	93.76	93.77	88.23
MGCNet	Mult	ResNet	93.73	93.31	93.52	88.01
CMFNet	Mult	ResNet	92.82	91.74	92.28	85.83
CMGFNet	Mult	ResNet	91.89	91.26	91.57	84.75
SFANet	Mult	ResNet	94.15	93.52	93.81	88.76
FTransUNet	Mult	ResNet	93.09	92.58	91.45	86.20
MFMINet	Mult	ResNet	95.12	93.64	93.97	89.96

**Table 2:** The quantitative evaluation results of comparison methods and the complexities metrics are based on the Vaihingen dataset, and the best result for each column is highlighted in bold

Methods	Modality	Background	Car	Tree	Low Vegetation	Building	Imperious Surface	mIoU%
DeepLabV3+	Uni	96.89	77.73	86.43	83.80	94.31	82.54	86.95
PSPNet	Uni	97.07	70.81	86.15	82.85	93.74	81.43	85.34
SegNet	Uni	96.17	72.07	84.79	81.22	93.08	80.86	84.70
FCN-8s	Uni	96.39	75.51	83.73	79.59	92.84	80.30	84.76
CMTFNet	Uni	97.53	**82.97**	87.11	84.87	94.77	83.35	88.23
MGCNet	Mult	97.54	82.02	86.80	84.42	94.52	82.74	88.01
CMFNet	Mult	94.28	79.34	84.91	81.70	93.98	80.78	85.83
CMGFNet	Mult	96.10	74.75	84.22	80.25	93.16	80.03	84.75
SFANet	Mult	97.12	80.17	88.75	86.79	95.24	84.46	88.76
FTransUNet	Mult	92.83	78.91	86.37	83.85	93.23	82.01	86.20
MFMINet	Mult	**97.62**	81.23	**90.57**	**88.73**	**96.04**	**85.62**	**89.96**

MFMINet demonstrates outstanding performance across nearly all categories when compared to other methods. By leveraging elevation information from nDSM data, our model is adept at accurately segmenting similar objects, particularly in the tree and low-value cover categories. MFMINet outperforms SFANet by 1.82% in the tree category and by nearly 2.00% in the low-value cover category, emphasizing the importance of utilizing nDSM data for enhanced semantic segmentation of HRRSI. Moreover, our method achieves an impressive 96.04% mIoU in the building category, indicating that MFMINet successfully addresses the challenges of capturing edge details and reducing noise in nDSM for HRRSI semantic segmentation.

**Figure 7:**
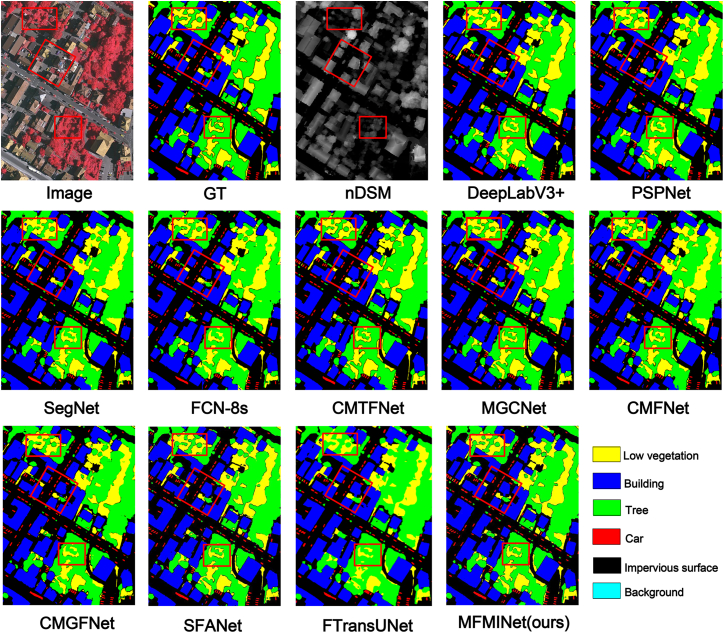
The qualitative evaluation results of Vaihingen dataset On the Vaihingen dataset, visual comparisons show the input (IRRG and nDSM), predictions from several SOTA methods, the ground truth (GT), and our MFMINet result for different scenes. MFMINet demonstrates more accurate object boundaries and better discrimination of similar classes (e.g., trees versus low vegetation) compared to other methods.

To illustrate the superiority of our method over other prevalent techniques, we present a visual comparison in Figure 7. The figure clearly shows that MFMINet yields superior segmentation results. It effectively discriminates between spatially proximate and similar categories such as trees and low vegetation cover. Furthermore, even with smaller target objects like cars, MFMINet demonstrates precise segmentation capabilities. However, in terms of edge delineation, MGCNet and CMTFNet exhibit slightly better performance than our model.

#### Experiments on Potsdam dataset

In the final phase of our evaluation, we benchmarked our proposed method against other SOTA approaches on the ISPRS Potsdam dataset. The outcomes are detailed in Tables 3 and 4, where our model attains SOTA performance across multiple metrics, securing the highest mIoU score of 88.24%. Notably, our method excels in the categories of cars, trees, and low vegetation, showcasing its proficiency in capturing intricate spatial details and accurately identifying various land cover types.

**Table 3:** Results evaluated by recall (%), precision (%), F1 (%), and mIoU (%) on the Potsdam dataset

Method	Modality	Backbone	Recall	Precision	F1	mIoU
DeepLabV3+	Uni	ResNet	91.40	91.31	91.35	84.20
PSPNet	Uni	ResNet	90.48	90.93	90.70	83.13
SegNet	Uni	VGG	90.57	90.87	90.72	83.17
FCN-8s	Uni	VGG	88.84	89.06	88.95	80.31
CMTFNet	Uni	ResNet	90.98	91.00	90.99	83.62
MGCNet	Mult	ResNet	93.73	90.46	90.10	82.15
CMFNet	Mult	ResNet	91.54	91.74	91.64	84.31
CMGFNet	Mult	ResNet	89.93	89.83	89.88	81.82
SFANet	Mult	ResNet	93.15	92.57	92.91	86.77
FTransUNet	Mult	ResNet	90.96	92.40	91.56	85.05
MFMINet	Mult	ResNet	94.13	93.45	93.69	88.24

**Table 4:** The quantitative evaluation results of comparison methods and the complexities metrics are based on the Potsdam dataset, and the best result for each column is highlighted in bold

Methods	Modality	Background	Car	Tree	Low Vegetation	Building	Imperious Surface	mIoU%
DeepLabV3+	Uni	82.33	84.20	77.68	81.84	95.47	83.71	84.20
PSPNet	Uni	80.55	83.19	76.89	80.74	94.81	82.58	83.13
SegNet	Uni	79.73	83.66	76.31	80.85	95.02	83.47	83.17
FCN-8s	Uni	73.97	82.65	73.42	77.44	93.52	80.84	80.31
CMTFNet	Uni	80.52	84.73	76.92	81.08	95.07	83.41	83.62
MGCNet	Mult	78.13	83.66	74.76	78.91	95.21	82.23	82.15
CMFNet	Mult	81.74	84.91	78.08	81.52	95.87	83.71	84.31
CMGFNet	Mult	77.06	83.03	75.06	78.96	95.03	81.78	81.82
SFANet	Mult	86.37	85.89	81.51	85.03	96.48	85.26	86.77
FTransUNet	Mult	80.35	84.75	79.69	84.40	95.27	85.86	85.05
MFMINet	Mult	**87.54**	**86.93**	**83.57**	**87.26**	**97.72**	**86.43**	**88.24**

In the automobile category, our method achieves an impressive mIoU score of 86.93%, which is more than 1% point higher than the second-best method, SFANet. This underscores the superiority of our approach over similar multimodal methods and highlights MFMINet’s ability to effectively integrate complementary information, enhancing the model’s semantic learning capabilities. Furthermore, the categories with high interclass similarity, such as trees and low vegetation, our method still delivers exceptional results, with mIoU scores of 83.57% and 87.26%, respectively. These scores are more than 2% higher than those of the closest competitors, clearly demonstrating our method’s efficacy in distinguishing between objects with strong interclass consistency.

**Figure 8:**
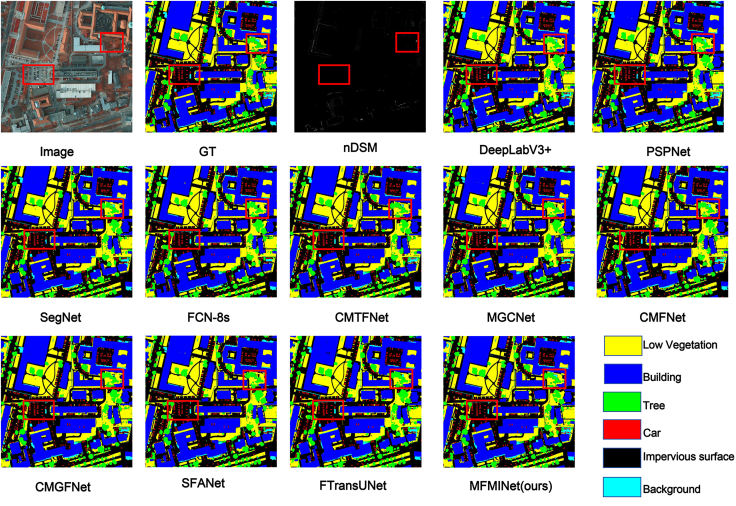
The qualitative evaluation results of Potsdam dataset On the Potsdam dataset, visual comparisons show the input (IRRG and nDSM), predictions from several SOTA methods, the ground truth (GT), and our MFMINet result for different scenes. MFMINet provides more complete object coverage and accurately segments small objects (e.g., cars) and challenging categories (e.g., trees).

Figure 8 provides a qualitative comparison of the semantic segmentation results of various methods on the Potsdam dataset. It is evident that the MFMINet model excels in segmenting the tree and low vegetation classes, offering more comprehensive segmentation outlines. In the car category, which constitutes a relatively small proportion of the pixel count, MFMINet successfully locates and delineates the shapes of vehicles with greater clarity. Overall, when compared to the other methods, our proposed approach delivers superior segmentation outcomes.

#### Computational complexity

We have conducted a comparison of the model parameters (Params), the number of Giga Floating-point Operations (GFLOPs), and inference speed of MFMINet against other models, with the findings presented in Table 5. By examining the performance indicators of various segmentation models, we can identify the pronounced benefits of our proposed approach across several aspects. While MFMINet’s GFLOPs performance is average, its parameter count of 133.09M is moderate and appropriate, striking a healthy balance between performance and feasibility. Moreover, MFMINet requires only 0.39 h per training epoch, which is more time-efficient than its counterparts, such as MGCNet’s 1.63 h and CMFNet’s 1.11 h, enhancing its practicality, particularly in environments with limited resources. Although FTransUNet and MGCNet exhibit stronger GFLOPs, the high parameter count of 208.36M in MGCNet and 160.88M in FTransUNet may result in elevated storage and computational expenses. MFMINet, however, maintains a lower computational complexity while facilitating an efficient training procedure, making it more competitive for real-world deployment. This demonstrates MFMINet’s superior efficiency and judicious resource utilization.

**Table 5:** Comparison of computational complexity and efficiency of different deep learning models on GTX 1080ti GPU

Methods	GFLOPs	Params(M)	h/Epoch
DeepLabV3+	32.53	47.93	0.36
PSPNet	14.84	46.71	0.32
SegNet	40.32	29.45	0.33
FCN-8s	10.88	51.95	0.45
CMTFNet	8.57	30.07	0.42
MGCNet	47.59	208.36	1.63
CMFNet	78.26	98.50	1.11
CMGFNet	38.84	85.22	0.65
SFANet	16.52	108.36	0.82
FTransUNet	25.38	160.88	0.75
MFMINet	19.11	133.09	0.39

### Ablation studies

To assess the impact of the core components within our proposed MFMINet, we performed a series of ablation studies. The details of these experiments and their outcomes are presented in Table 6, focusing on the four modules of MFMINet. The presence of a symbol denotes that a particular module was kept, while its absence signifies that the module was excluded from the architecture. All other experimental parameters, such as the loss function and optimizer, were maintained in line with the fully intact MFMINet configuration. The findings reveal that each module is instrumental in boosting the segmentation capabilities of MFMINet, with the full model yielding the highest performance. Additionally, the degradation observed in performance across all metrics when the DSAM, SAM, FEM, or MCFM module is omitted serves to underscore the vital role of each component in the network’s efficacy.

**Table 6:** The impact of different module combinations on model performance

DSAM	FEM	MCFM	SAM	mIoU	F1
–	✓	✓	✓	88.38	93.61
✓	–	✓	✓	88.15	93.52
✓	✓	–	✓	88.73	93.83
✓	✓	✓	–	88.56	93.74
✓	✓	✓	✓	89.96	93.97

#### The effect of MCFM

**Figure 9:**
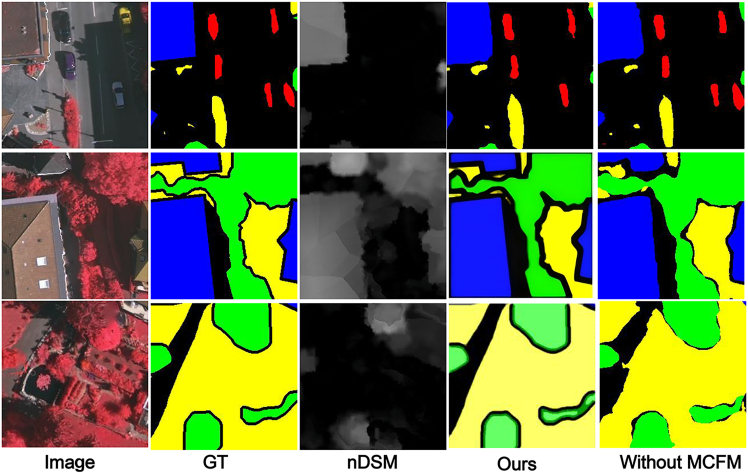
MFMINet without MCFM The figure compares the GT, prediction without MCFM, and full MFMINet prediction. Removing MCFM leads to noticeably coarser segmentation and failure in capturing small objects, underscoring its role in effective fusion.

We replaced MCFM with simple element-wise addition for IRRG and nDSM cross-modal fusion, using a point-wise convolutional layer to adjust the number of channels before feature addition. Figure 9 shows that without noise filtering and effective fusion between the cross-modal features, resulting in rough segmentation and failure to extract small objects.

#### The effect of SAM

**Figure 10:**
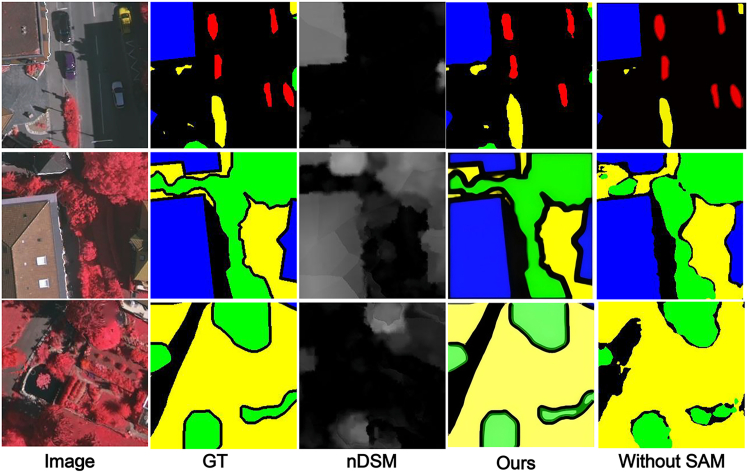
MFMINet without SAM The comparison among GT, prediction without SAM, and full model shows that removing SAM results in blurred boundaries and region adhesion, highlighting its importance for global context modeling.

We replaced the SAM with direct feature passing from the fusion stage to the DSAM module, eliminating the global spatial dependency modeling step. Figure 10 shows that without global context modeling and long-range spatial dependency capture, the network fails to establish coherent spatial relationships across distant regions, resulting in blurred and rough segmentation boundaries, partial region adhesion, and decreased detection performance for small objects.

#### The effect of DSAM

**Figure 11:**
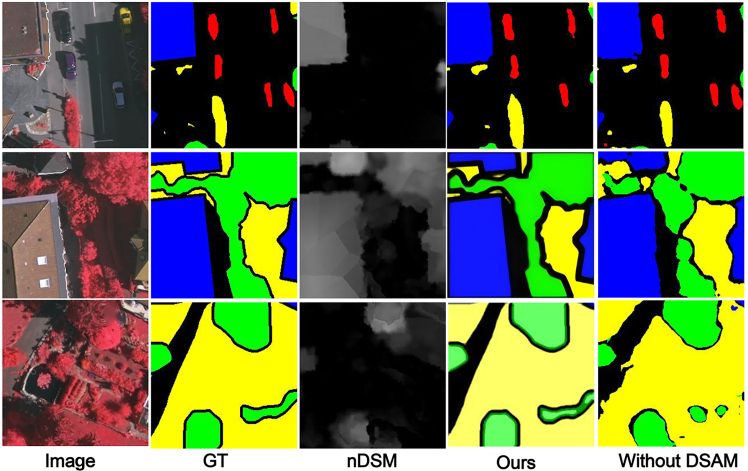
MFMINet without DSAM The images show the GT, prediction without DSAM, and full model result. The absence of DSAM reduces the model’s ability to leverage multiscale context, leading to less coherent segmentation in complex areas.

We use simple feature fusion instead of DSAM, and Figure 11 shows that without effective extraction of semantic information from deeper features, it can result in a model that struggles to effectively model global semantic features.

#### The effect of FEM

**Figure 12:**
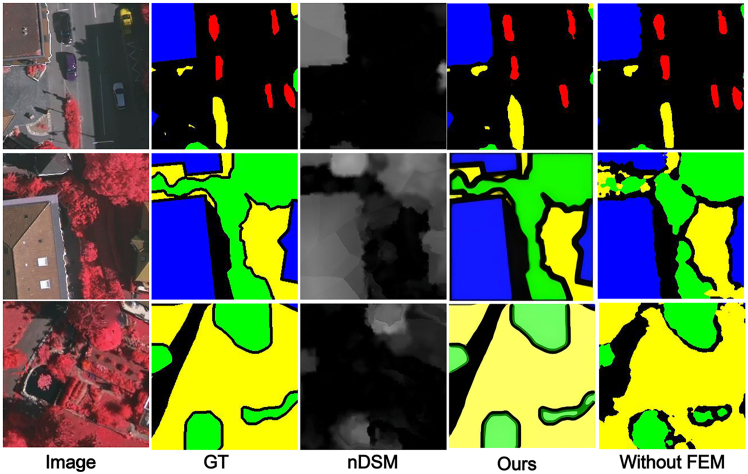
MFMINet without FEM The panel displays the GT, prediction without FEM, and full model output. Replacing FEM with a simple fusion causes loss of spatial details and reduces accuracy in distinguishing similar objects, proving FEM’s effectiveness in feature refinement.

We use a simple convolutional layer in the decoder to replace the FEM in order to fuse the features from the encoder output, and use upsampling to resize the feature map. Figure 12 shows that when simple feature fusion is performed by ignoring the differences between the layers of features, it results in a model that loses spatial detail information and thus fails to accurately discriminate between targets with similarities.

### Conclusions

We have proposed the MFMINet model, aiming to enhance the performance of multimodal semantic segmentation for HRRSI. The core of our model is the MCFM, which ingeniously combines high-level semantic features with low-level spatial details for more accurate segmentation.

Additionally, we innovatively introduce the dual spatial awareness module (DSAM), which utilizes a dual-path processing strategy to fully exploit global multiscale contextual information, guiding the decoding process. To enhance global context modeling capabilities, we incorporate a SAM between the multimodal feature fusion and DSAM stages, which effectively captures long-range spatial dependencies and refines the fused RGB-DSM features before multiscale processing. In the decoder part, we have developed the FEM, which intelligently selects transformer blocks for narrow channels and CNN blocks for wide channels, followed by point-wise convolution for optimal feature integration. This adaptive approach effectively integrates features from different levels and removes redundant information, further refining the precision of the semantic segmentation map. Experimental validation on two benchmark datasets has shown that MFMINet exhibits outstanding performance across all performance metrics.

Although the MFMINet model has brought a new research perspective to the field of HRRSI semantic segmentation, we recognize that optimizing the number of model parameters and enhancing the feature extraction capability for small target objects remain key directions for our future work. We will continue to explore and aim to achieve more efficient and precise semantic segmentation technology for remote sensing images.

1. A network model MFMINet for cross-modal semantic segmentation of IRRG images using nDSM is proposed, which retains the advantages of IRRG images and effectively fuses complementary information to facilitate the model’s learning of semantic information. Experiments on the International Society for Photogrammetry and Remote Sensing (ISPRS) Vaihingen and Potsdam datasets show that the method achieves good semantic segmentation results.
2. We propose an MCFM module to fully integrate nDSM and IRRG multimodal information. Additionally, we introduce SAM module to capture long-range spatial dependencies and enhance global context modeling of the fused features. Furthermore, we use DSAM module to extract multiscale contextual features by dual-path interaction and incorporating a channel attention mechanism, which establishes relationships between features at different scales.
3. We propose a FEM module for fusing the features extracted by the encoder and decoder for efficient feature aggregation. The FEM intelligently selects between transformer blocks for narrow channels (≤128) and CNN blocks for wide channels (>128), followed by point-wise convolution for optimal channel information integration.

### Limitations of the study

Despite the promising performance achieved by MFMINet on the ISPRS Vaihingen and Potsdam datasets, several limitations of the present study should be acknowledged.

First, although the proposed method demonstrates strong qualitative performance in segmenting small objects such as cars, the quantitative evaluation mainly relies on overall metrics (e.g., mIoU and F1-score across all classes). While class-wise mIoU for the “Car” category is reported, a more targeted and in-depth analysis specifically designed for small object segmentation—such as instance-level metrics or size-aware evaluation protocols—has not been conducted. Given the severe class imbalance and the limited pixel proportion of small objects in high-resolution remote sensing datasets, future work will focus on adopting more specialized evaluation strategies to further validate the robustness of MFMINet in small-object scenarios.

Second, the experimental validation in this study is limited to two urban benchmark datasets with similar sensing modalities and scene characteristics. Although MFMINet achieves consistent improvements on both Vaihingen and Potsdam datasets, its cross-scene and cross-dataset generalization ability has not yet been thoroughly investigated. Differences in spatial resolution, land-cover distribution, sensor characteristics, and scene complexity—such as those present in datasets like UAVid or LoveDA—may affect the model’s performance. Therefore, the current results may not fully reflect the generalization capability of MFMINet under more diverse or unseen conditions. Extensive validation on additional datasets and more complex scenarios will be an important direction for future research.

Finally, MFMINet introduces multiple attention-based and fusion modules, which inevitably increase the model’s parameter size and architectural complexity. Although the computational efficiency remains acceptable in the current experimental setting, further optimization and lightweight design will be necessary to facilitate large-scale deployment and real-time applications.

## Resource availability

### Lead contact

Requests for further information and resources should be directed to and will be fulfilled by the lead contact, Qiongqiong Hu (huqiongqiong@qhu.edu.cn).

### Materials availability

This study did not generate new unique reagents.

### Data and code availability

• This study used the publicly available benchmark datasets provided by ISPRS: the Vaihingen dataset and the Potsdam dataset.
  ○ Vaihingen dataset has been deposited at Zenodo and is publicly available as of the date of publication at Zenodo: https://doi.org/10.5281/zenodo.18017380.
  ○ Potsdam dataset has been deposited at Zenodo and is publicly available as of the date of publication at Zenodo: https://doi.org/10.5281/zenodo.18026857.
• All original code has been deposited at Zenodo and is publicly available at Zenodo: https://doi.org/10.5281/zenodo.18016407 as of the date of publication.
• Any additional information required to reanalyze the data reported in this paper is available from the lead contact upon request.

## Acknowledgments

This work was supported by the Kunlun Talents·High-end Innovation and Entrepreneurship Talent Project of Qinghai Province (Grant No. k9925096) and the Postgraduate Research and Practice Innovation Project of Qinghai University in 2025 (Grant No. 2025-GMKY-48). We would like to extend our sincere gratitude to Dr. Qiongqiong Hu for her invaluable guidance and constructive suggestions throughout the course of this research. We acknowledge the International Society for Photogrammetry and Remote Sensing (ISPRS) for providing the Vaihingen and Potsdam benchmark datasets. We are also grateful to the students for their technical assistance in code debugging and experiments, and to our colleagues for their insightful feedback during internal seminars. Finally, we wish to especially thank the anonymous reviewers for their thoughtful comments, which have greatly contributed to the improvement of this manuscript.

## Author contributions

Conceptualization, F.W. and J.C.; methodology, J.C. and J.F.; investigation, Q.H., Y. Zhu, and Y. Zhang; data curation, J.C.; writing – original draft, F.W. and J.C.; writing – review and editing, Q.H. and J.C.; resources, Y. Zhang and J.F.; supervision, Q.H. and Y. Zhu; project administration, Q.H.

## Declaration of interests

The authors declare no competing interests.

## Declaration of generative AI and AI-assisted technologies in the writing process

During the preparation of this work, the authors used Chat-GPT 4.0 with caution in order to improve language and readability of some paragraphs. After using this tool/service, the authors reviewed and edited the content as needed and take full responsibility for the content of the publication.

## STAR★Methods

### Key resources table

**Table:** 

REAGENT or RESOURCE	SOURCE	IDENTIFIER
**Deposited data**		

ISPRS Vaihingen Dataset	ISPRS Commission III, Working Group 4 (ISPRS WG III/4)	Zenodo: https://doi.org/10.5281/zenodo.18017380
ISPRS Potsdam Dataset	ISPRS Commission III, Working Group 4 (ISPRS WG III/4)	Zenodo: https://doi.org/10.5281/zenodo.18026857

**Software and algorithms**		

Visual Studio Code	Microsoft	https://code.visualstudio.com/
Python 3.10.11	Python Software Foundation	https://www.python.org/
PyTorch 2.0.1	Meta AI	https://pytorch.org/
Code	This paper	Zenodo: https://doi.org/10.5281/zenodo.18016407

**Other**		

NVIDIA GeForce GTX 1080 Ti GPU	NVIDIA	N/A

### Experimental model and study participant details

This study does not involve human participants, animal models, or biological materials. All experiments were conducted on publicly available high-resolution remote sensing datasets for computational semantic segmentation.

#### Datasets

We evaluated MFMINet on two benchmark datasets from the ISPRS 2D Semantic Labeling Contest.

ISPRS Vaihingen dataset
Content: 33 aerial image tiles covering a village area in Vaihingen, Germany
Optical modality: IRRG orthophotos
Elevation modality: normalized Digital Surface Models (nDSM)
Spatial resolution: 9 cm ground sampling distance
Classes: Impervious surfaces, Building, Low vegetation, Tree, Car, Background
Tile size: variable, average 2494 × 2064 pixels
ISPRS Potsdam dataset
Content: 38 aerial image tiles covering a city district of Potsdam, Germany
Optical modality: IRRG orthophotos
Elevation modality: nDSM
Spatial resolution: 5 cm ground sampling distance
Classes: Impervious surfaces, Building, Low vegetation, Tree, Car, Background
Tile size: 6000 × 6000 pixels

#### Data preprocessing and partitioning

Due to the large tile size, both datasets were cropped into patches of 256 × 256 pixels using a non-overlapping sliding window. This resulted in:

Vaihingen: 10,113 training and 2,398 validation patches
Potsdam: 40,627 training and 10,157 validation patches
To enhance training diversity, data augmentation included horizontal flipping, random rotation, and noise addition.

### Method details

#### Model architecture: MFMINet

The overall structure of MFMINet is shown in Figure 1, which consists of an encoder and a decoder. The input images of MFMINet are a three-channel IRRG image and a single-channel nDSM image (elevation values in the nDSM image are converted to grey-scale images). For efficiency, we use the ResNet-50 structure to extract multiscale features from the IRRG images. Since the nDSM image contains less feature information and does not require too many extracted features, we use the lighter ResNet-34 structure to extract the multiscale features of the nDSM image. Each ResNet-50 and ResNet-34 contains five convolutional blocks: Conv1, Res-2, Res-3, Res-4, Res-5. The output of the i-th convolutional block used to extract features from the IRRG image is denoted as *Ri* (i=1, 2, 3, 4, 5). The output of the ith convolutional block used to extract features from the nDSM image is denoted as *Di* (i=1, 2, 3, 4, 5).

For encoding, we use four MCFM modules to fuse cross-layer and cross-modal features. After feature fusion, SAM is introduced to model long-range spatial dependencies in the fused features, enhancing global context understanding before multi-scale feature extraction. Then, the DSAM module is used to extract the multi-scale context as guidance information. For decoding, we employ a Feature Enhancement Module (FEM) that intelligently selects between Transformer blocks for narrow channels (≤ 128) and CNN blocks for wide channels (> 128), followed by point-wise convolution for channel information integration. This adaptive approach gradually fuses high-level semantic information with low-level spatial detail information, and finally obtains the final semantic segmentation result map.

#### Multimodal Cross-layer fusion module (MCFM)

Since the resolution of remote sensing images is significantly larger than that of natural images, the receptive field of the model needs to be improved. Although, the pooling layer in the encoder enlarges the receptive field, it loses the low-level spatial detail information. In addition, for multimodal data, it is necessary to make full use of complementary features for data fusion. However, basic operations, including element-wise multiplication or feature-level fusion, can inadvertently increase noise in the extracted features. Therefore, we adopt MCFM (Figure 2) for data fusion, since it utilizes only a limited number of downsampling operations, which helps mitigate noise accumulation. To speed up the computational efficiency, we use point-wise convolution on the input data first, thus reducing the number of parameters of the model and adjusting the number of channels. For the IRRG branch, we establish two parallel convolutional layer branches to more accurately extract the important regions of IRRG cross-layer features. For the nDSM branch, we also designed two parallel branches. However, unlike the IRRG branch, we employ a sigmoid function to suppress less informative features.

Specifically, for the i-th MCFM module, it takes *R*_*i*_, *R*_*i*+1_, and *D*_*i*+1_ as input features. We apply max pooling to *R*_*i*_ to adjust its feature map size to match *R*_*i*+1_. Through two convolutional blocks, we obtain feature maps $R_{1}^{RGB}$ and $R_{2}^{RGB}\in R^{C\times H\times W}$, respectively. Then, we use element-wise multiplication to avoid redundancy from addition and to extract feature information from $R_{1}^{RGB}$ and $R_{2}^{RGB}$, resulting in *F*_*R*_, as follows:

(Equation 5) $$R_{1}^{RGB}=CBR_{3\times 3}\left(CBR_{1\times 1}\left(MaxPool\left(R_{i}\right)\right)\right)$$

(Equation 6) $$R_{2}^{RGB}=CBR_{3\times 3}\left(CBR_{1\times 1}\left(R_{i+1}\right)\right)$$

(Equation 7) $$F_{R}=R_{1}^{RGB}⊙R_{2}^{RGB}$$

*D*_*i*+1_ is processed as follows to generate *F*_*D*_:

(Equation 8) $$F_{D}=Conv_{1\times 1}\left(D_{i+1}\right)⊙Sigmoid\left(CBR_{3\times 3}\left(D_{i+1}\right)\right)$$

Finally, we concatenate the data from the IRRG and nDSM branches and pass it through a convolutional layer with a kernel size of 1 to adjust the number of channels. Subsequently, Dropout with a rate of 0.7 is applied for regularization to enhance the model’s generalization capability and mitigate overfitting.

(Equation 9) F=Concat(FD,FR)

(Equation 10) $$output=DropOut\left(CBR_{1\times 1}\left(F\right)\right)$$

#### Self-attention module (SAM)

To enhance the global context modeling capability of fused multi-modal features, we introduce a Self-Attention Module (SAM) between the feature fusion stage and the multi-scale feature extraction stage(as shown in Figure 3). This module enables the network to capture long-range spatial dependencies, which is crucial for accurate semantic segmentation in complex remote sensing scenes.

Given the input feature map $X\in R^{B\times C\times H\times W}$ from the fused RGB-DSM features, the module first generates query (Q), key (K), and value (V) representations through 1×1 convolutions with a reduction ratio r=8:

$$Q=Conv_{q}\left(X\right)\in R^{B\times C/r\times H\times W}$$

$$K=Conv_{k}\left(X\right)\in R^{B\times C/r\times H\times W}$$

$$V=Conv_{v}\left(X\right)\in R^{B\times C\times H\times W}$$

The spatial attention matrix is computed as:

(Equation 11) $$A=Softmax\left(Q^{T}\times K\right)\in R^{HW\times HW}$$

where each element represents the attention weight between spatial locations. The attended features are obtained by:

(Equation 12) $$Z=V\times A^{T}$$

The final output incorporates a learnable parameter *γ* and residual connection:

(Equation 13) $$Y=\gamma \cdot Conv_{out}\left(Z\right)+X$$

where *γ* is initialized to 0 to ensure training stability. SAM is strategically placed after multi-modal feature fusion and before the DSAM module, allowing the network to refine fused features and enhance contextual information for subsequent multi-scale feature extraction. The reduction ratio significantly reduces computational complexity from O(C^2^HW) to O((C/r)^2^HW), making it feasible to apply self-attention to high-dimensional feature maps without excessive computational overhead.

#### Dual Spatial Awareness Module (DSAM)

Multi-scale contextual information is crucial for solving the semantic ambiguity problem. Therefore, we propose DSAM (Figure 4) to further enhance the semantic relationships in multi-scale contextual information. Specifically, DSAM connects R5 and D5 as input data and extracts semantic information using a convolutional layer with a convolutional kernel size of 3 × 3. Then, six parallel branches are used to generate multi-scale features. The first branch employs global average pooling to compress high-dimensional features into 1D vectors. A following 1D convolution then models cross-channel interactions, whose output (normalized by a sigmoid function) is used to perform element-wise multiplication with the input features, thereby highlighting salient information and reducing redundancy. The next four branches use dilated convolution (kernel size 3×3, dilation rates 1, 2, 5 and 11 respectively) to reduce the number of parameters in the model to speed up the computational efficiency of the model. The last branch uses global average pooling and adjusts the number of channels by a convolution with a kernel size of 1×1, then fuses the features from the four dilated convolutional branches, and the fused feature maps are summed with the feature maps from the first branch by channel adjustment.

#### Feature Enhancement Module (FEM)

In order to effectively improve the model’s ability to perceive multi-scale information, we propose a Feature Enhancement Module (FEM) in the decoder, which is shown in Figure 5. Unlike traditional feature enhancement approaches that apply uniform processing to all feature maps, FEM intelligently selects the optimal processing strategy based on the channel dimensions of input features. The FEM employs a channel-adaptive mechanism that automatically determines the processing pathway: For narrow-channel features (C ≤128 ): The module utilizes a Transformer Block that leverages multi-head self-attention to capture global contextual relationships:

(Equation 14) $$Attention\left(Q,K,V\right)=Softmax\left(\frac{QK^{T}}{\sqrt{d_{k}}}\right)V$$

(Equation 15) $$MultiHead\left(Q,K,V\right)=Concat\left({\text{head}}_{1},\ldots ,{\text{head}}_{h}\right)W^{O}$$

where ${\text{head}}_{i}=\text{Attention}\left(QW_{i}^{Q},KW_{i}^{K},VW_{i}^{V}\right)$.

For wide-channel features (C >128): The module employs a CNN Block with residual connections to maintain computational efficiency while preserving local feature extraction capability:

(Equation 16) $$Y_{\text{CNN}}={\text{Conv}}_{2}\left(\text{ReLU}\left(\text{BN}\left({\text{Conv}}_{1}\left(X\right)\right)\right)\right)+X$$

Both processing branches are followed by point-wise convolution for channel information integration:

(Equation 17) $$X_{\text{out}}=\text{PWConv}\left(\text{ProcessedFeature}\right)$$

(Equation 18) $$X_{\text{final}}=X_{\text{out}}+X_{\text{residual}}$$

where ProcessedFeature is the output from either the Transformer Block or CNN Block, and PWConv represents the 1×1 point-wise convolution operation. The FEM first performs channel dimension evaluation on the input feature map, then routes the features through the appropriate processing branch. The Transformer branch excels at modeling long-range dependencies for features with fewer channels, while the CNN branch maintains computational efficiency for high-dimensional features. The threshold of 128 channels was empirically determined based on a trade-off between computational efficiency and feature expressiveness. Transformer blocks are computationally expensive at higher channel dimensions due to their quadratic complexity with respect to channel size. Through preliminary experiments and supervisory guidance, we observed that applying Transformer blocks to features with channel dimensions ≤128 yields stable performance improvements without significant computational overhead, whereas CNN blocks are more efficient and effective for wider feature representations (>128). This adaptive design ensures optimal feature representation while controlling computational complexity. The module includes residual connections that preserve original feature information and enable stable gradient flow during training.

#### Loss function

We employ a loss function that combines Focal Loss (FL) and Dice Loss (DL) to optimize the final output feature map, as shown in Equation 19. The formulas for DL and FL are detailed in Equations 20 and 21, respectively. In Equation 20, *p* represents the actual label value, while $\hat{p}$ denotes the model’s predicted value. In Equation 21, the modulation factor ${\left(1-p_{t}\right)}^{\gamma }$ is introduced to diminish the loss contribution from samples that are easy to distinguish, regardless of whether they belong to the foreground or background category. A higher value of *p*_*t*_ indicates that the sample is easier to identify, resulting in a smaller modulation factor. Additionally, *α*_*t*_ is utilized to balance the loss contributions from positive and negative samples. Furthermore, the number of buildings in the two datasets utilized differs significantly from the number of background samples. To mitigate the negative impact of sample imbalance, we incorporate the Dice Loss function.

(Equation 19) $$L_{total}=\frac{FL+DL}{2}$$

(Equation 20) $$DL=1-\frac{2\left|p\cap \hat{p}\right|}{\left|p\right|+\left|\hat{p}\right|}$$

(Equation 21) $$FL\left(p_{t}\right)=-\alpha_{t}{\left(1-p_{t}\right)}^{\gamma }\;\log\left(p_{t}\right)$$

#### Input preprocessing

Prior to network input, optical IRRG images were normalized by dividing pixel values by 255.0, scaling the intensity range to [0,1]. The normalized IRRG images were then used as three-channel inputs to the optical encoder. The nDSM data were treated as single-channel inputs representing elevation information and were spatially resized in a manner consistent with the corresponding optical images during both data augmentation and evaluation. This ensured pixel-wise alignment between the two modalities throughout training and inference.

#### Training configuration

In this study, the experiments were conducted on a system running Ubuntu 20.04, equipped with an NVIDIA GTX1080Ti GPU and 12 GB of RAM. The model was implemented using PyTorch 2.0.1 and Python 3.10.11. For optimization, the Adam optimizer was employed with a weight decay of 0.0001 and an initial learning rate of 5e-4. The training process was set for a maximum of 100 iterations. In each iteration, a batch of 16 samples was used for training. One complete iteration was defined as processing all training images in the dataset. The model was trained using a composite loss function combining Dice loss and Focal loss, with the Focal loss parameters set to *α* = 0.75 and *γ* = 2 to mitigate class imbalance.

#### Data augmentation

Training-time augmentation consisted of random scaling with aspect ratio jitter and random horizontal flipping (p = 0.5). During validation and evaluation, a deterministic resizing and padding strategy was adopted to preserve the original aspect ratio. All transformations were jointly applied to optical images, nDSM images, and ground-truth labels.

#### Inference and prediction

During evaluation and inference, the trained model was applied to the validation set, where each image patch was processed independently. The predicted semantic labels were saved as single-channel grayscale masks. For large-scale inference, a directory-based batch prediction mode was employed, in which the model iterates over all input images in a specified folder and writes the corresponding output masks to a designated directory.

### Quantification and statistical analysis

#### Evaluation metrics

Model performance was quantitatively evaluated using standard semantic segmentation metrics computed at the pixel level for each class, including Precision, Recall, F1 score, and mean Intersection over Union (mIoU). Among these, mIoU was selected as the primary evaluation metric. It is calculated as the average of the ratio of intersection to union between predicted and ground-truth pixels across all classes, which effectively indicates how well each category is segmented. Evaluation was conducted on the validation set by comparing the predicted segmentation masks with the corresponding ground-truth grayscale labels.

#### Evaluation protocol

The validation procedure was conducted as follows. First, image identifiers were loaded from a predefined validation split. For each identifier, the corresponding IRRG image and nDSM data were processed together by the trained model to generate and save a segmentation mask. For evaluation, model performance was quantified by computing the mIoU through comparison of the predicted masks with the ground-truth labels.

#### Reproducibility

All experiments used the same dataset split lists and identical training configurations to ensure fair comparisons.

The superiority of MFMINet was validated through comparative experiments with published models (MGCNet,^43^ CMGFNet,^42^ FTransUNet,^45^ etc.) on the same datasets and under identical experimental conditions. As illustrated in Tables 1, 2, 3, and 4.

Ablation experiments were performed by removing individual modules (MCFM, SAM, DSAM, FEM) and evaluating the performance degradation, demonstrating the contribution of each component. As illustrated in Table 6.

#### Software for analysis

Evaluation metrics were computed using Python scripts based on PyTorch and NumPy. Results were summarized and visualized using Matplotlib/OpenCV pipelines implemented in the codebase.
